# Adsorption and Catalytic Activity of Gold Nanoparticles in Mesoporous Silica: Effect of Pore Size and Dispersion Salinity

**DOI:** 10.1021/acs.jpcc.1c09573

**Published:** 2022-01-26

**Authors:** Yingzhen Ma, Gergely Nagy, Miriam Siebenbürger, Ravneet Kaur, Kerry M. Dooley, Bhuvnesh Bharti

**Affiliations:** † Cain Department of Chemical Engineering, Louisiana State University, Baton Rouge, Louisiana 70803, United States; ‡ Neutron Scattering Division, Oak Ridge National Laboratory, Oak Ridge, Tennessee 37831, United States; § Center for Advanced Microstructures and Devices, Louisiana State University, Baton Rouge, Louisiana 70806, United States; ∥ Life and Physical Science Department, Ivy Tech Community College of Indiana, Valparaiso, Indiana 46360, United States

## Abstract

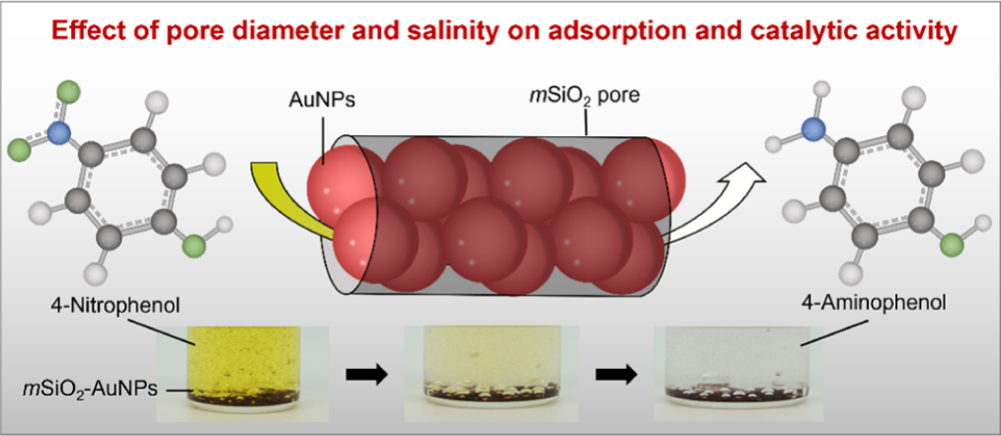

The assembled state of nanoparticles (NPs) within porous matrices plays a governing role in directing their biological, electronic, and catalytic properties. However, the effects of the spatial confinement and environmental factors, such as salinity, on the NP assemblies within the pores are poorly understood. In this study, we use adsorption isotherms, spectrophotometry, and small-angle neutron scattering to develop a better understanding of the effect of spatial confinement on the assembled state and catalytic performance of gold (Au) NPs in propylamine-functionalized SBA-15 and MCM-41 mesoporous silica materials (*m*SiO_2_). We carry out a detailed investigation of the effect of pore diameter and ionic strength on the packing and spatial distribution of AuNPs within *m*SiO_2_ to get a comprehensive insight into the structure, functioning, and activity of these NPs. We demonstrate the ability of the adsorbed AuNPs to withstand aggregation under high salinity conditions. We attribute the observed preservation of the adsorbed state of AuNPs to the strong electrostatic attraction between oppositely charged pore walls and AuNPs. The preservation of the structure allows the AuNPs to retain their catalytic activity for a model reaction in high salinity aqueous solution, here, the reduction of *p*-nitrophenol to *p*-aminophenol, which otherwise is significantly diminished due to bulk aggregation of the AuNPs. This fundamental study demonstrates the critical role of confinement and dispersion salinity on the adsorption and catalytic performance of NPs.

## 1 Introduction

The stability and spatial distribution of metallic nanoparticles (NPs) in aqueous solution is the key in harvesting their unusual properties in the fields of pharmaceuticals, biosensors, and catalysis.^1−5^ Due to the high surface energy and large surface area-to-volume ratio, the dispersed state of metallic NPs in aqueous solution is thermodynamically unfavorable leading to their spontaneous aggregation.^6−8^ A common strategy to stabilize NPs in aqueous medium is via physical/chemical adsorption of surfactants or ligands onto NPs imparting a kinetic barrier against the aggregation.^9−11^ However, the surfactants or ligands can significantly alter the physical and chemical properties of the NPs, especially their catalytic performance in aqueous media.^12,13^ Recent studies have proposed to overcome such a limitation by immobilizing catalytic NPs within an inert porous matrix.^14−16^ Here, the NPs are either synthesized within the confined pore space or the presynthesized NPs are physically/chemically adsorbed onto the pore walls. The immobilization of NPs in porous material is anticipated to preserve the NP stability in extreme environments such as high salinity media while retaining the catalytic activity, albeit reduced due to mass transport limitations (discussed later). However, there is a lack of understanding of the impacts of pore diameter, particle concentration, and solvent conditions on the assembled state of NPs within the porous material. This limitation exists due to the lack of our ability to effectively characterize NP assemblies in situ using traditional spectroscopic techniques, where the inert matrix interferes. In this study, we overcome these challenges by combining NP adsorption isotherm studies with small-angle neutron scattering (SANS), which enables the identification of the in situ state of NPs. We investigate the binding of model gold (Au) NPs within the mesopores of the inert silica matrix (SBA-15 and MCM-41) and quantify the structure and catalytic performance of the NPs in high salinity aqueous medium.

The AuNPs are often utilized in confined environments such as tissue matrix, porous catalytic supports, and nanotubes.^17−20^ In order to optimize the efficiencies of the nanomaterials, it is critical to uncover and understand the adsorbed state of AuNPs in the confined spaces and the impacts of environmental parameters such as pH and salinity on their properties. Previously, it has been reported that the confinement of porous materials can break the structural symmetry of the adsorbing nanomaterial and drive the formation of unusual structures such as zigzag, helices, and NP multilayers.^21−24^ Additionally, such pore-confined NPs can provide control over the selectivity in the product of the catalyzed reaction.^25−27^ Despite the unusual properties of metallic NPs in pores, the surface interactions governing the formation of complex nanoassemblies in confined spaces are poorly understood.

AuNPs are widely used in biomedicine, environmental, and industrial applications primarily because of their unusual optoelectronic and catalytic properties.^28,29^ One of the major challenges faced in using the AuNPs in real environments is stabilization of these particles under extreme pH and salinity conditions. Recently, the partial coating of gold nanorods with silica has been proposed as an alternative which improves the colloidal stability of the core–shell structure and enables the chemical reduction of phenol to nontoxic products.^18^ However, the synthesis of such a Au-silica core–shell structure requires complex steps and can be anticipated to suffer from limitations similar to that of silica NPs, which are prone to aggregation in a high salinity environment. Here, we show that these limitations can be overcome by immobilizing AuNPs in the pores of SBA-15 and MCM-41 mesoporous silica materials. We investigate the effect of pore diameter and the presence of electrolyte (NaCl) on the equilibrium amount of NPs adsorbed in the porous materials and on their catalytic activity.

## 2 Materials and Methods

### 2.1 Materials

Following are the details of the chemicals used in the study. The list provides the supplier of the chemicals and their purity: tetraethyl orthosilicate (TEOS, Sigma-Aldrich, ≥ 99%), HCl (5 N, VWR), ammonium fluoride (NH_4_F, Alfa Aesar, ≥ 98%), decane (Sigma-Aldrich, ≥ 99%), hexane (Sigma-Aldrich, ≥ 99%), hexadecyltrimethylammonium bromide (CTAB, Sigma-Aldrich, ≥ 99%), gold chloride trihydrate (HAuCl_4_·3H_2_O, Sigma-Aldrich, ≥ 99.9%), sodium citrate dihydrate (C_6_H_5_Na_3_O_7_·2H_2_O, Sigma-Aldrich, ≥ 99%), sodium borohydride (NaBH_4_, Sigma-Aldrich, ≥ 98%), (3-aminopropyl)triethoxysilane (APTES, Sigma-Aldrich, ≥ 98%), and poly(ethylene oxide)-poly(propylene oxide)-poly(ethylene oxide) triblock copolymer (Pluronic P123, Sigma-Aldrich, ≥ 98%)

### 2.2 Synthesis of AuNPs

**Figure 1:**
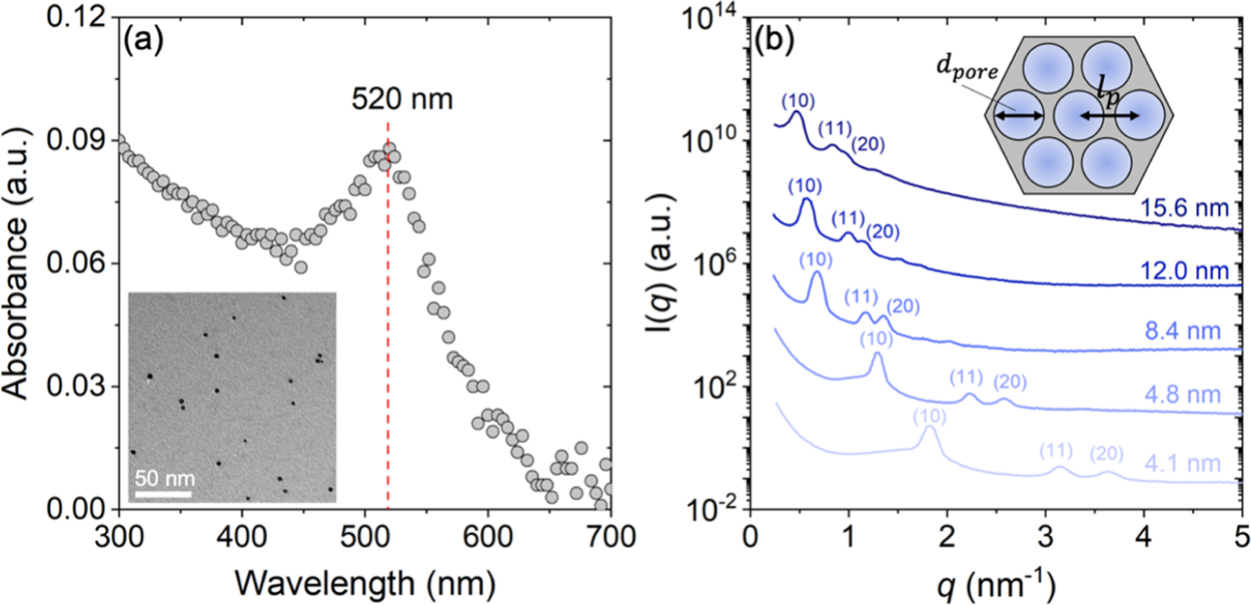
(a) UV–vis spectra of AuNPs synthesized by chemical reduction of HAuCl_4_. The maximum absorbance was observed at a wavelength of 520 nm, which is the characteristic surface plasmon resonance of AuNPs with 3–5 nm diameter. This result is in agreement with particle sizes obtained by TEM (inset), which shows that the average diameter of AuNPs in bulk solution is ∼4 nm. (b) SAXS profiles of *m*SiO_2_ with various *d*_pore_. The curves are shifted by a constant factor of 100 for better visualization. The inset schematic shows the 2D *hcp* pore lattice with lattice parameter and pore diameter. The numbers in the parentheses represent the miller indices of the corresponding Bragg peak.

The AuNPs were synthesized by reducing HAuCl_4_·3H_2_O using NaBH_4_ in the presence of sodium citrate solution. In a typical synthesis, 0.01 g of HAuCl_4_ and 0.0075 g of sodium citrate were mixed with 100 mL of deionized water, and then, 3 mL of 0.1 M freshly prepared NaBH_4_ solution was added to the mixture under constant stirring at 20 °C.^30^ The mixture transformed to pink immediately after adding NaBH_4_ solution, indicating the formation of AuNPs. During the synthesis, sodium citrate was used as a capping agent which induced a negative charge (zeta potential = −41.4 mV at pH 6) on the particles and provided kinetic stability to the AuNPs in low salinity aqueous solutions via electrostatic double layer repulsion. The AuNPs were characterized for their size using UV–vis spectrophotometry, as shown in Figure 1a. The peak at wavelength 520 nm is the signature of surface plasmon resonance from ∼4 nm AuNPs,^30^ which is confirmed by transmission electron microscopy (TEM) shown in the inset of Figures 1a and S1, which provided the average size of 4 ± 0.4 nm.

### 2.3 Synthesis of Mesoporous Silica

The model mesoporous silica materials MCM-41 and SBA-15 with pore diameters (*d*_pore_) of 4.1, 4.8, 8.4, 12.0, and 15.6 nm were synthesized using previously reported methods.^31−35^ These silica materials had cylindrical nanopores arranged in a two-dimensional (2D) hexagonal closed packed (*hcp*) lattice.^36^

#### 2.3.1 MCM-41 Silica with *d*_pore_ = 4.1 nm

The mesoporous silica was synthesized using CTAB as the structure-directing template in the presence of the silica precursor TEOS under alkaline conditions. Here, 1.0 g of CTAB was mixed with 480 mL of deionized water, and 1.4 mL of 5 M NaOH was added into the solution. The mixture was kept at 80 °C for 2 h under constant stirring and reflux. The product was filtered, washed with deionized water, and dried at 70 °C in the oven. Then, the organic template was removed from the silica materials by calcination at 550 °C for 4 h.

#### 2.3.2 SBA-15 Silica Materials with *d*_pore_ = 4.8, 8.4, 12.0, and 15.6 nm

SBA-15 materials with different pore diameters were synthesized following the established method.^32−35^ For *d*_pore_ = 4.8 nm pore diameter, SBA-15 is synthesized by dissolving 2.84 g of P123 in 90 g of deionized water and 48 g of 5 M HCl solution. The mixture is equilibrated at 35 °C for 2 h, and then, 8.0 g of TEOS was added dropwise and reacted for 20 h at 35 °C. Stirring was maintained throughout. For *d*_pore_ = 8.4 nm, TEOS, H_2_SO_4_, H_2_O, and P123 were dissolved in deionized water in the molar ratio of 1TEOS/5.9H_2_SO_4_/323H_2_O/0.017P123, kept at 40 °C for 5 h, and then aged at 105 °C for 20 h in an autoclave. In the case of *d*_pore_ = 12.0 nm pore diameter, 2.4 g of P123 was dissolved in 84 mL of HCl aqueous solution (1.3 N) at 25 °C for 6 h. Then, 0.027 g of NH_4_F was added to the mixture and kept for 20 min. At this stage, 14.3 g of decane and 5.5 mL of TEOS were premixed and then dropwise added into the mixture, kept at 30 °C for 20 h, and then transferred into a convection oven for further reaction (no stirring) at 100 °C for 48 h. The SBA-15 with *d*_pore_ = 15.6 nm pore size was synthesized using a similar method as 12.0 nm SBA-15, but with premixed 8.45 g of hexane with 5.5 mL of TEOS added to the mixture of P123, HCl, and NH_4_F. The mixture was kept at 15 °C for 20 h. The four silica material products were filtered, washed with deionized water, dried at 100 °C, and calcined at 550 °C for 5 h to remove the surfactant template.

### 2.4 Propylamine Functionalization of Mesoporous Silica

We chemically modified the pore walls of the silica materials with propylamine groups and abbreviate these mesoporous silicas as *m*SiO_2_ hereon.^37^ The propylamine functionalization of the mesoporous silica was achieved by reacting with APTES in acidic solution. In a typical synthesis, 1.0 g of the prepared silica material was mixed with 4.0 mL of acetic acid and 6.0 mL of deionized water. The mixture was equilibrated at 80 °C for 30 min under reflux, and then, 0.12 mL of APTES was added. This mixture continued to react for 16 h under reflux, and the product was filtered, washed five times with 100 mL of deionized water, and then dried at 60 °C for 24 h. The amine-functionalized pore walls of the *m*SiO_2_ were positively charged at pH 6 due to protonation of the amine group; thus, an electrostatic attraction existed between the negatively charged AuNPs and *m*SiO_2_ at pH 6.^37^ The direct measurement of zeta potential of *m*SiO_2_ porous particles is nontrivial because of their large size, polydispersity, and aggregation. However, the zeta potential can be assumed equal to propylamine-functionalized silica nanospheres, which is +35.7 mV at pH 6. The silica nanospheres were synthesized using an identical experimental procedure as reported in our previous work.^37^ The 2D *hcp* structure of pores in *m*SiO_2_ led to the appearance of characteristic Bragg peaks in small-angle X-ray scattering (SAXS) experiments, as shown in Figure 1b. The pore diameter and lattice parameter of the MCM-41 and SBA-15 silica materials used in the study are shown in Table 1. Note that the pore diameter is determined using nitrogen gas adsorption, as shown in Figure S2.

**Table 1:** Characterization of *m*SiO_2_ Using Nitrogen Gas Adsorption and SAXS

s. no.	*d*_pore_ (nm)	lattice parameter, *lp* (nm)
1	4.1 ± 0.1	4.4 ± 0.3
2	4.8 ± 0.1	5.6 ± 0.3
3	8.4 ± 0.3	10.7 ± 1.4
4	12.0 ± 0.3	12.9 ± 2.0
5	15.6 ± 0.2	16.5 ± 3.1

## 3 Results and Discussion

### 3.1 Adsorption of AuNPs in Mesoporous *m*SiO_2_

**Figure 2:**
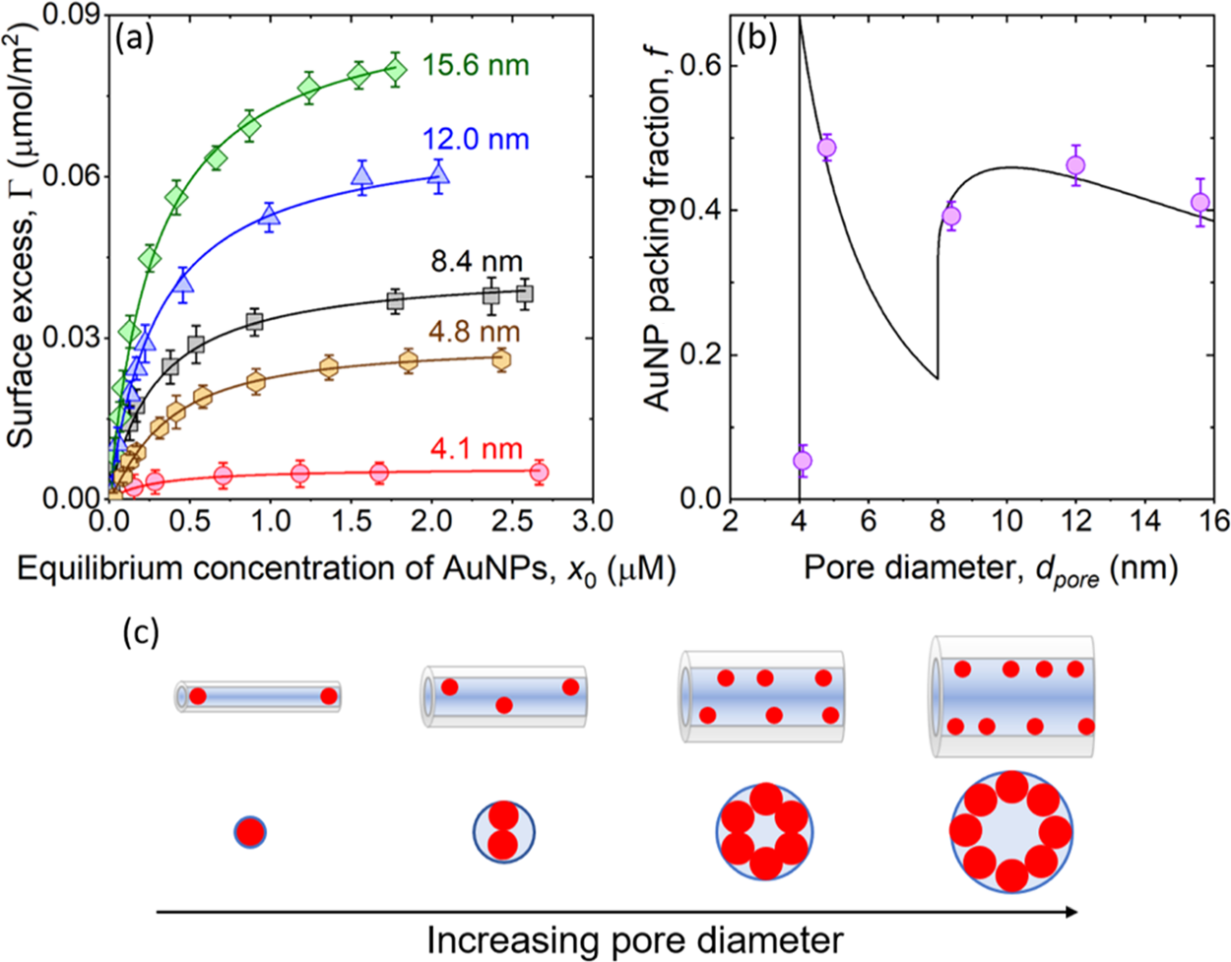
(a) Adsorption isotherms for AuNPs binding to the cylindrical nanopores of *m*SiO_2_ with increasing *d*_pore_ = 4.1, 4.8, 8.4, 12.0, and 15.6 nm. The scattered points are the experimental data and solid lines represent the best fit to the data using the Langmuir model given using eq 1. (b) Theoretical curves (black line) and experimental data points (circles) of the maximum AuNP packing fraction in *m*SiO_2_ pores as a function of pore diameter. (c) Schematic representing the change in packing of the AuNPs in the silica matrix with increasing *d*_pore_ as predicted using the SVC model.

To understand the effect of *d*_pore_ of *m*SiO_2_ on the adsorption behavior, the amount of AuNPs adsorbed (Γ) in the pores of *m*SiO_2_ is measured using the solvent depletion method.^33,38^ Here, the aqueous dispersion containing AuNPs at pH 6 is equilibrated with a fixed amount of *m*SiO_2_ for 24 h. After equilibration, the *m*SiO_2_ with adsorbed AuNPs are separated from the dispersion using centrifugation for 30 min at 18,000*g* (see the Supporting Information for settling time calculations). The concentration of the unadsorbed AuNPs in a solvent is determined using its characteristic spectrophotometric absorbance value at 520 nm (Figures 1a, and S3). The amount of NPs adsorbed per unit surface area is calculated using the equation Γ = (*x* – *x*_0_)*V*/*mS*, where *x* is the initial concentration of the AuNPs in the mixture, (*x* – *x*_0_) is the concentration of AuNPs adsorbed in *m*SiO_2_, *V* is the volume of aqueous solution, *S* is the specific surface area, and *m* is the mass of *m*SiO_2_ added to the solution. The isotherms for the adsorption of AuNPs in *m*SiO_2_ of increasing *d*_pore_ are shown in Figure 2a. We find that the maximum amount of NPs adsorbed per unit surface area of *m*SiO_2_ increases with increasing *d*_pore_. Here, we use the Langmuir model for quantitative analysis of the experimentally obtained isotherms. Mathematically, the Langmuir adsorption model is represented as^39^
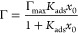
1

Here, Γ is the amount of AuNPs adsorbed into *m*SiO_2_ pores, Γ_max_ is the maximum surface excess of the AuNPs, *x*_0_ is the equilibrium concentration of AuNPs in bulk, and *K*_ads_ is the equilibrium adsorption constant which is the measure of binding energy of NPs to the pore walls. Based on the analysis of our experimental data, we find that the maximum amount of AuNPs adsorbed on *m*SiO_2_ increases nearly by an order of magnitude upon increasing *d*_pore_ from 4.1 to 15.6 nm. Note that the surface excess is normalized to the surface area, and the observed increase in the adsorbed amount of AuNPs with increasing *d*_pore_ is due to the reduced geometric barrier for AuNPs penetrating the *m*SiO_2_ matrix.^40,41^ At *d*_pore_ comparable to the diameter of the AuNPs, adsorption observed is significantly lowered due to the physical barrier for NPs accessing the pore space. The small amount of AuNPs adsorbed at *d*_pore_ = 4.1 nm is likely due to the binding of the AuNPs on the *m*SiO_2_ matrix exterior to the pores.

The analysis of the adsorption isotherms gives a binding affinity of *K*_ads_ ∼ 3.5 mM^–1^ corresponding to the adsorption free energy ∼ −29.6 kJ/mol, which is similar to previously reported values for surfactant adsorption onto silica surfaces.^42,43^ Note that the binding affinity values of AuNPs remain nearly independent of the *d*_pore_, highlighting that the nature of the chemical species controlling the adsorption remains unchanged, that is, −NH_3_^+^ (from propylamine) of *m*SiO_2_ and −COO^–^(from citrate) of AuNPs.

The changes in the maximum amount of AuNPs loaded in the pore space with increasing *d*_pore_ can be compared quantitatively using the maximum packing fraction (*f*). The value of *f* can be determined as *f* = *nV*_part_/*V*_pore_, where *n* is the total number of AuNPs adsorbed, *V*_part_ is the volume of AuNPs which is (4/3)π*R*^3^, where *R* is the mean radius of AuNPs, and *V*_pore_ is the total volume of pores. The value of *n* is determined experimentally using Γ_max_ as *n* = *mS*Γ_max_*N*_A_, and *V*_pore_ = (*mS*/π*d*_pore_*l*) × π*d*_pore_^2^*l*/4 = *mSd*_pore_/4, where *l* is the length of pores. Therefore, the value of *f* can be obtained from experimental adsorption isotherms as *f* = 16π*R*^3^Γ_max_*N*_A_/3*d*_pore_. Assuming AuNPs as hard spheres, the value of *f* can be calculated theoretically using a geometrical model proposed by Sang, Vinu, and Coppens (SVC).^44^ The nonlinear change in the maximum pore-filling fractions with *d*_pore_ as estimated using experimental adsorption isotherms (circles) and SVC model (line) is shown in Figure 2b. Note that the theoretically predicted value of *f* = 0.67 at *d*_pore_ = 2*R* is due to the assumed spherical shape of the particles adsorbing in tubular pores. This contrasts with the calculations performed by Meissner et al.^45^ where the authors used cylindrical shape to represent the protein molecules adsorbing in the tubular pores and obtained *f* = 1 at *d*_pore_ = 2*R*. The experimental value of *f* first shows a rapid increase followed by a slight decrease with increasing *d*_pore_, which agrees with the predictions of the SVC geometric model showing step changes in the values of *f*, as depicted in Figure 2c. The large *f* values highlight that the AuNPs penetrate the whole pore space, instead of blocking or plugging them at the pore entrance. Such a penetration of AuNPs can be achieved by surface diffusion of the particles, as reported previously for flat surfaces.^46^ Note that the electrostatic double layer repulsion between negatively charged AuNPs would contribute to the interparticle separation and corresponding experimental *f*-values.

### 3.2 Effect of the Added Electrolyte on Adsorption

**Figure 3:**
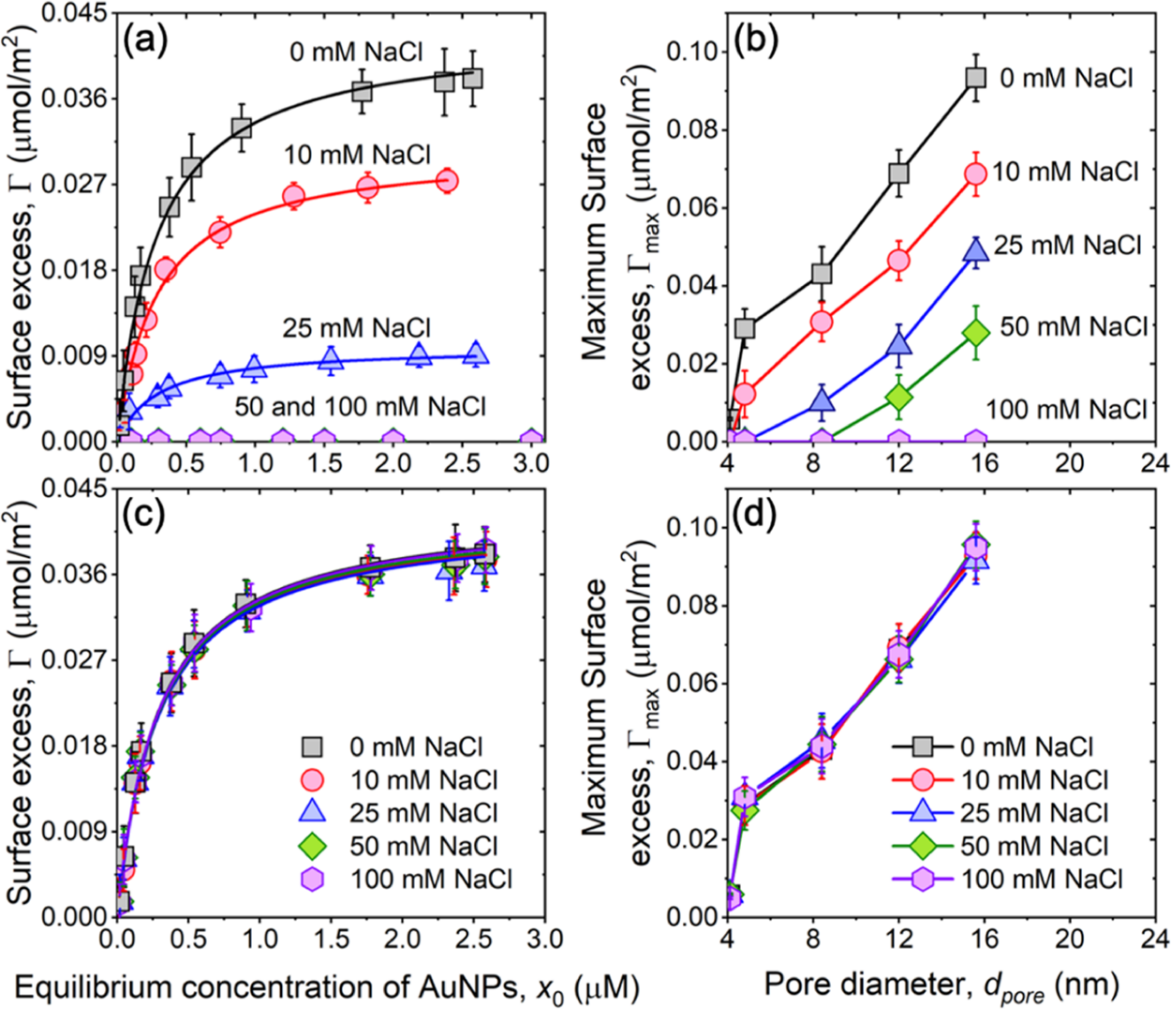
(a) Preadsorption: adsorption isotherms of AuNPs in *m*SiO_2_ with *d*_pore_ = 8.4 nm upon the addition of NaCl. Here, the electrolyte was added prior to the initiation of the adsorption process. (b) Maximum surface excess of AuNPs in *m*SiO_2_ when the increasing amount of NaCl was added prior to the NP adsorption process with *d*_pore_ = 4.1, 4.8, 8.4, 12.0, and 15.6 nm. The observed decrease in the maximum surface excess with the addition of NaCl is due to the aggregation of AuNPs which inhibits the adsorption of NPs in the tubular pores. (c) Postadsorption: adsorption isotherms of AuNPs on 8.4 nm *m*SiO_2_ with increasing concentration of NaCl. The electrolyte is added after loading the AuNPs in *m*SiO_2_. (d) Maximum surface excess of AuNPs in *m*SiO_2_ when increasing amount of NaCl was added after the NP adsorption process with *d*_pore_ = 4.1, 4.8, 8.4, 12.0, and 15.6 nm. The constant maximum surface excess of AuNPs highlights no significant desorption of AuNPs from the pore wall which results from the strong electrostatic attraction between negatively charged AuNPs and positively charged *m*SiO_2_.

The interparticle repulsions between negatively charged AuNPs can be screened by the addition of electrolyte in the aqueous medium. Here, we use NaCl as a model 1:1 electrolyte to screen the electrostatic interactions and investigate the effect of the electrolyte concentration on the colloidal stability and adsorption of AuNPs to *m*SiO_2_. We measure the adsorption isotherms of AuNPs on *m*SiO_2_ with *d*_pore_ = 4.1, 4.8, 8.4, 12.0, and 15.6 nm in the presence of 10, 25, 50, and 100 mM NaCl (Figures 3a–d, S4 and S5). The adsorption isotherms are measured by adding the electrolyte at two different adsorption stages: (1) preadsorption: NaCl is added prior to the adsorption of AuNPs in *m*SiO_2_; and (2) postadsorption: NaCl is introduced to the dispersion containing *m*SiO_2_ with adsorbed AuNPs. In the case where the electrolyte is added to the AuNP dispersion prior to the adsorption, the Γ_max_ is dependent on the amount of added salt which decreases with increasing NaCl concentration (Figures 3a and S4). The observation can be attributed to the larger size of the aggregates formed by AuNPs at higher NaCl concentration. In stark contrast, the AuNPs remain bound to the pore walls when the electrolyte is added after the completion of the adsorption process (Figure 3c).

**Figure 4:**
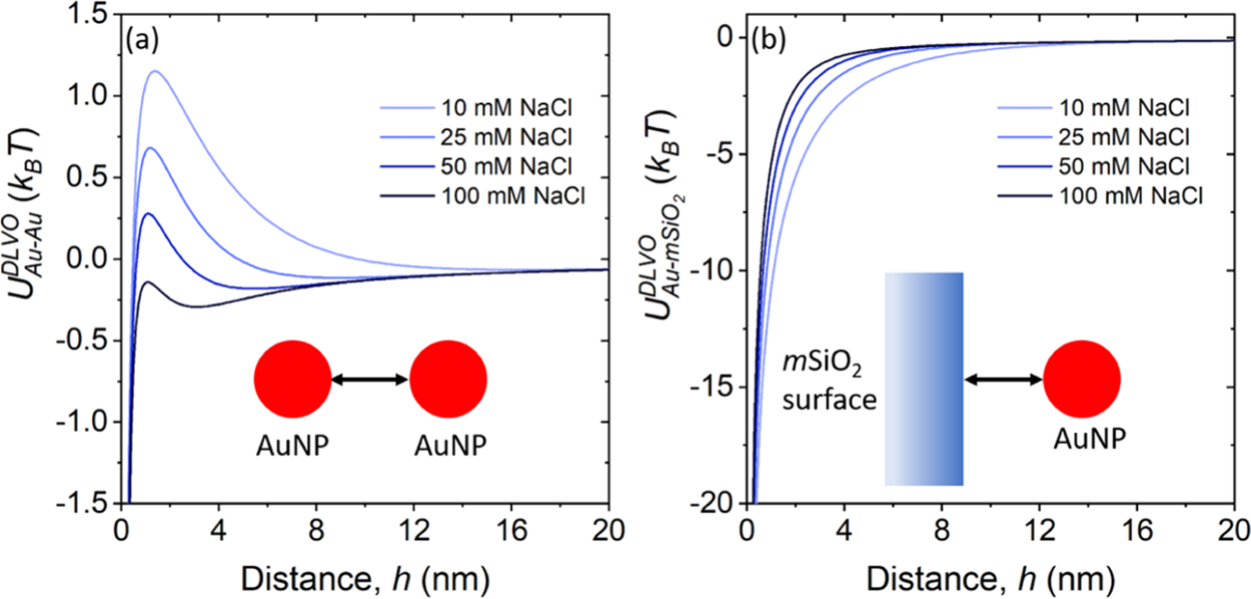
DLVO interaction energy between a (a) pair of AuNPs and (b) AuNP and *m*SiO_2_ surface in the presence of 10, 25, 50, and 100 mM NaCl. The repulsion between AuNPs is reduced with increasing concentration of NaCl and results in aggregation of the NPs. The attraction between the oppositely charged AuNPs and *m*SiO_2_ is not significantly impacted upon the addition of NaCl. The insets are schematics of the interaction of AuNP–AuNP and *m*SiO_2_–AuNP, respectively.

The electrostatic attraction between the oppositely charged *m*SiO_2_ pore wall and AuNPs drives the system to a free energy minimum, which is not altered by the addition of salt (Figure 3d). While in the case of AuNPs dispersed in aqueous medium, the addition of electrolyte screens the electrical double layer repulsions between the NPs leading to the dominance of van der Waals interaction and aggregation. The net interaction energy between a pair of colloidal particles and a particle and a flat substrate can be estimated using Derjaguin–Landau–Vervey–Overbeek (DLVO) theory (see the Supporting Information for details). The net interaction energy (*U*^DLVO^) between a pair of AuNPs and a AuNP and *m*SiO_2_ surface is shown in Figure 4. The interaction energy calculations show that the repulsion between AuNPs is screened upon the addition of NaCl leading to aggregation of the NPs (Figure 4a). However, no significant effect of the addition of salt on interaction energy is observed for oppositely charged AuNPs and *m*SiO_2_ (Figure 4b). Note that the electronegativity of the protonated aminopropyl functional group (−C_3_H_6_NH_3_^+^) is lower than that of Na^+^, that is, the ion–pair association strength of Na^+^ is weaker, which restricts the desorption of the AuNPs from the pore wall upon the addition of NaCl.^47^ It can be inferred that the adsorption free energy between the *m*SiO_2_ and AuNPs overwhelms the attraction energy between AuNPs in 100 mM NaCl aqueous solution and contributes to the stable state of AuNPs on the silica pore wall under high salinity conditions.

### 3.3 Characterization of AuNPs in *m*SiO_2_ Using SANS and TEM

**Figure 5:**
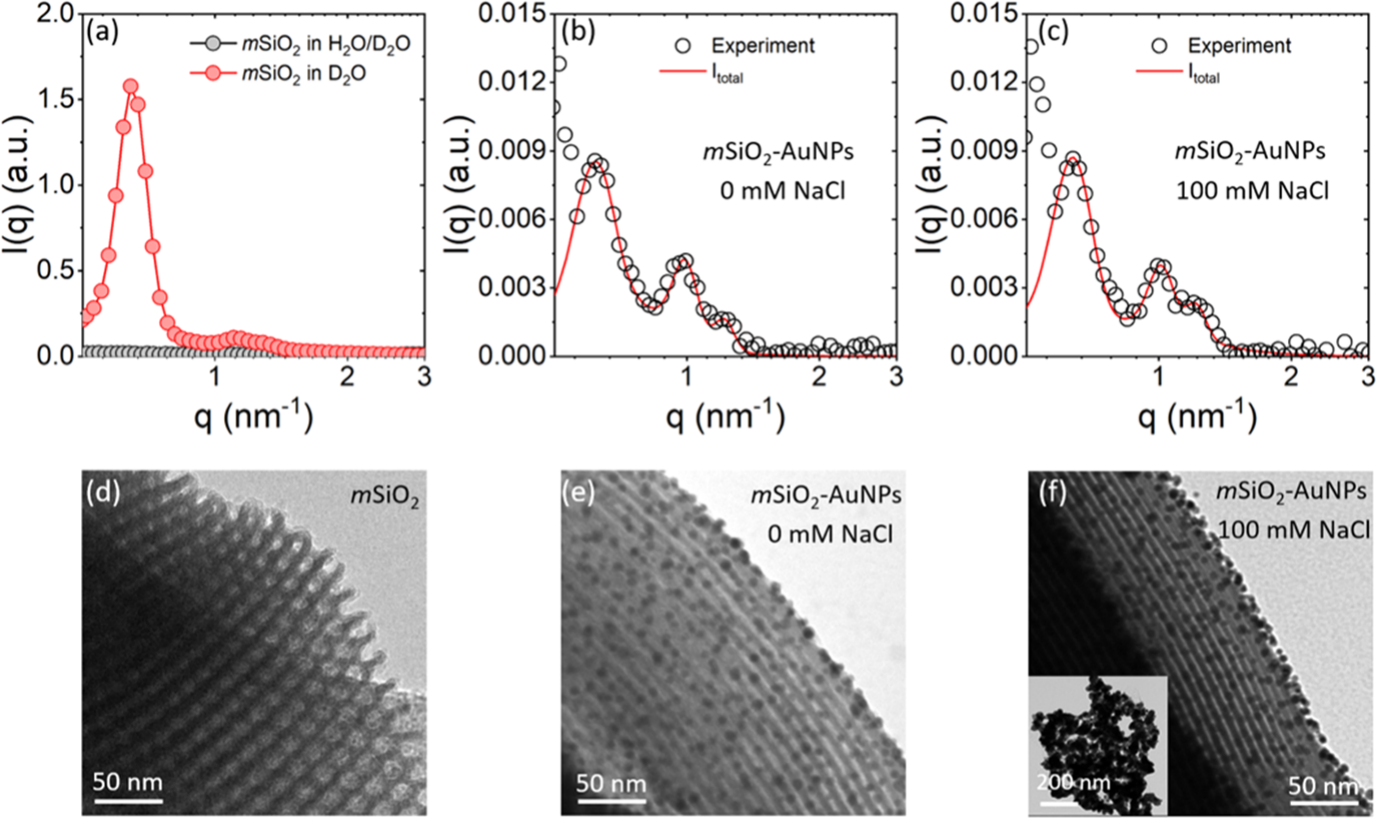
(a) SANS profiles for *m*SiO_2_ in D_2_O and the H_2_O/D_2_O mixture matching the scattering length density of *m*SiO_2_ (b,c) SANS profiles (circles) and the corresponding model fits (red lines) for AuNPs adsorbed on 8.4 nm pore diameter of *m*SiO_2_ under contrast-matched conditions in the presence of 0 and 100 mM NaCl solution. The increase in scattering intensity at *q* < 0.6 nm^–1^ in (b,c) can be attributed to the Porod’s scattering from the NPs adsorbed on the exterior surface of SBA-15 beads. (d–f) TEM images of *m*SiO_2_, *m*SiO_2_–AuNPs in DI water, and *m*SiO_2_–AuNPs in 100 mM NaCl aqueous solution, respectively. The inset in (f) shows the aggregates of AuNPs in bulk solvent in the presence of 100 mM NaCl.

The adsorption isotherms and the corresponding analysis provided an in-depth characterization of the amount of AuNPs loaded in *m*SiO_2_, but no information was obtained on the assembled state of AuNPs in the pores and corresponding impact of the addition of the electrolyte. We use SANS experiments to uncover the assembled state of AuNPs in *m*SiO_2_. The SANS experiments were performed using *m*SiO_2_ with *d*_pore_ = 8.4 nm in D_2_O and a 40:60 mixture of H_2_O/D_2_O matching the scattering length density (3.54 × 10^–4^ nm^–2^) of the silica matrix.^33^ The SANS for *m*SiO_2_ in D_2_O shows Bragg peak characteristic of the 2D *hcp* pore lattice of the silica matrix (Figure 5a,d). The Bragg peaks disappear when the experiment is performed in the H_2_O/D_2_O mixture (Figure 5a). We perform SANS on contrast-matched *m*SiO_2_ containing AuNPs at a concentration equivalent to 0.9Γ_max_ in the presence of 0 and 100 mM NaCl (Figure 5b,c). We find that upon the addition of AuNPs to contrast-matched *m*SiO_2_, the Bragg peaks reappear which is the signature of the presence of the NPs within the pore lattice (Figure 5a–c). Previously, it has been shown that the total scattering intensity (*I*_Total_) from mesoporous SBA-15/MCM-41 materials with adsorbed molecules/particles under silica contrast-matched conditions can be represented as^48^
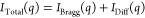
2where *q* is the scattering vector given as *q* = 4π/λ sinθ/2, λ is the wavelength, θ is the scattering angle, and *I*_Bragg_ and *I*_Diff_, respectively, are the Bragg and diffused scattering contributions to the total scattering. The Bragg scattering intensity is obtained by a Monte-Carlo simulation approach, where NPs are randomly distributed in the *m*SiO_2_ matrix and *I*_Diff_ is obtained using the Teubner-Strey model. Further details on the model and SANS data analysis are provided in the Supporting Information and our previous publication.^49^ We find that the theoretical model (lines in Figure 5b,c) effectively represents the experimental SANS data (circles in Figure 5b,c).

The presence of Bragg peaks for *m*SiO_2_ with adsorbed AuNPs despite being in a H_2_O/D_2_O mixture matching the scattering length density of silica confirms the presence of AuNPs adsorbed on the pore walls. Since the SANS experiments are performed at 0.9Γ_max_, all added AuNPs are adsorbed in the silica matrix. Based on our analysis of the diffused scattering contribution, we estimate a quasiperiodic distance of 6.9 nm between the AuNPs in the pore space (see the Supporting Information for details). We find that the quasiperiodic distance between AuNPs remains unchanged upon increasing the concentration of NaCl from 0 to 100 mM, indicating that the immobilized AuNPs retain their adsorbed spatially separated state in the high salinity aqueous solution. The retention of AuNPs within the pore upon the addition of NaCl highlights that the electrostatic attraction between the pore wall and AuNPs is not significantly screened, as shown in Figure 4b. The result is in agreement with the TEM images (Figure 5e,f), where AuNPs can be observed to be spatially separated and bound to the silica matrix instead of aggregating in the presence of NaCl. Note that TEM is performed on the dried suspension and SANS profiles are obtained in the presence of a solvent, highlighting that the AuNPs retain their adsorbed state upon drying the suspension.

### 3.4 Catalytic Activity of AuNPs

**Figure 6:**
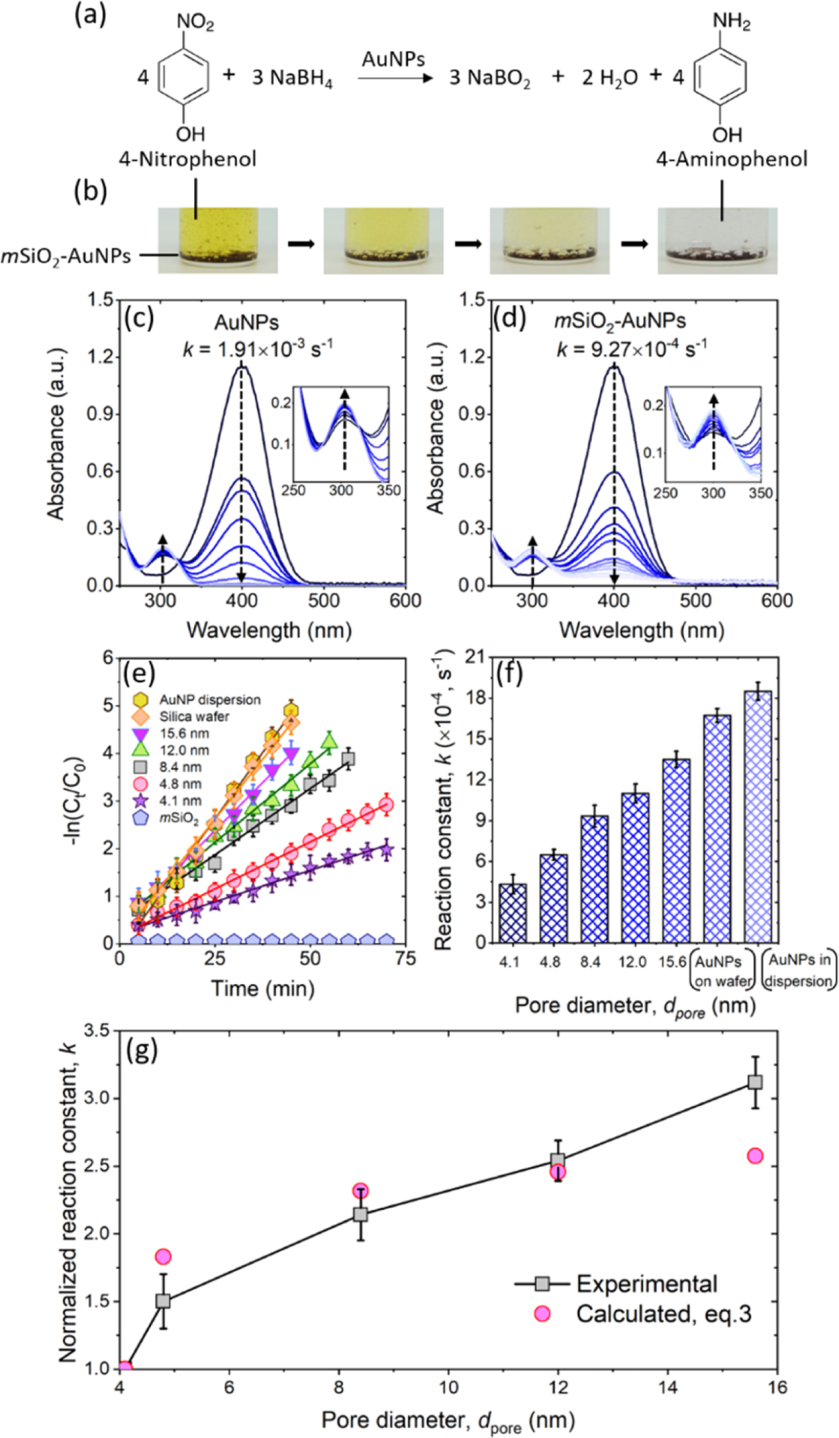
(a) Chemical equation of the 4-nitrophenol reduction reaction using AuNPs as a catalyst. (b) Photographs of the color change every 20 min for 4-nitrophenol aqueous solution in the presence of *m*SiO_2_ (*d*_pore_ = 8.4 nm) with adsorbed AuNPs. (c,d) UV–vis spectra and reaction constant of 4-nitrophenol reduction with both AuNPs and *m*SiO_2_–AuNP catalyst in DI water. Insets are the zoom-in plots of absorbance intensity in 250–350 nm wavelength. (e) Relationship between reaction time and −ln (*C*_*t*_/*C*_0_) is shown for various pore sizes of *m*SiO_2_ containing AuNPs, only *m*SiO_2_ matrix, AuNPs in dispersion, and AuNPs on a flat silica substrate. (f) Change in the reaction rate constant *k* for 4-nitrophenol reduction as a function of *d*_pore_ and AuNPs on the substrate and dispersed in bulk. (g) Reaction rate constants normalized to *k* of the 4.1 nm catalyst.

The spatial confinement imposed by the NP adsorption in mesopores and the colloidal stability of AuNPs in aqueous media are critical factors governing their catalytic activity.^50,51^ We use the reduction of 4-nitrophenol (yellow) to 4-aminophenol (colorless) by sodium borohydride (NaBH_4_) in the presence of AuNPs as a model reaction (Figure 6a) to investigate the changes in the catalytic performance of the NPs when immobilized in the pore space. We perform the reduction of 0.5 mM of 4-nitrophenol by 50 mM NaBH_4_ at 20 °C using AuNPs as a catalyst in following states: (A) dispersed in aqueous media, and (B) immobilized in *m*SiO_2_ with *d*_pore_ = 4.1, 4.8, 8.4, 12.0, and 15.6 nm (Figure 6b). The amount of AuNPs used in the measurements was equivalent to Γ = 0.7Γ_max_ = 0.002 μmol/m^2^ of *m*SiO_2_ with *d*_pore_ = 4.1 nm (Figure 2), that is, the identical number of AuNPs were present in all tested *m*SiO_2_ and all added NPs were solely present in their adsorbed state onto *m*SiO_2_. The spectrophotometric profiles obtained at various time steps during the reaction in the presence of AuNPs in the dispersed state and immobilized in 8.4 nm pore diameter *m*SiO_2_ are shown in Figure 6c,d. The spectrophotometric profiles show a peak at wavelength 400 nm, which is characteristic of the 4-nitrophenolate anion.^52^ The peak intensity gradually decreases with time indicating the decrease in the concentration of 4-nitrophenolate anions (Figure 6c,d). The observed decrease in the peak intensity at 400 nm wavelength is accompanied by an increase in absorbance peak intensity at 300 nm which is indicative of the gradual formation of 4-aminophenol.^52^ The observed changes in spectrophotometric profiles confirm the reduction of 4-nitrophenol to 4-aminophenol in the presence of AuNPs.

Since the concentration of NaBH_4_ overwhelmingly exceeds that of 4-nitrophenol, the reduction reaction can be represented using a pseudo-first-order reaction kinetics with respect to 4-nitrophenol.^53^ The rate equation for a first-order reaction is given as, *k*_*t*_ = −ln (*C*_*t*_/*C*_0_), where *k* is the reaction rate constant, *C*_0_ is the initial concertation of 4-nitrophenol, and *C*_*t*_ is the concentration after time *t*. Note that the absorbance values at wavelength 400 nm are converted to concentrations of 4-nitrophenol using a calibration curve, as shown in Figure S6. The changes in the values of −ln (*C*_*t*_/*C*_0_) with time for an identical number of AuNPs adsorbed in *m*SiO_2_ with *d*_pore_ = 4.1, 4.8, 8.4, 12.0, and 15.6 nm, for NPs suspended in solvent (DI water) and for only *m*SiO_2_, are shown in Figure 6e. The value of −ln (*C*_*t*_/*C*_0_) shows no change in the absence of AuNPs, but it increases linearly in all other cases. The rate constant is determined by the slope of each curve, which increases with increasing pore diameter (Figure 6f). The observed decrease in the rate constant for particles immobilized in the *m*SiO_2_ pores can be attributed to two major factors: (1) blocking of the active sites on AuNPs due to their adsorption onto the pore wall, which is confirmed by performing the reduction reaction for AuNPs immobilized on a flat surface showing a slightly lower rate constant than AuNPs in their suspended form (Figure 6e,f), and (2) limitations in the transport of reactants and products in and out of the spatially confined pore space. The reactions in the *m*SiO_2_ pores are clearly diffusion-limited, and factor (1) would be nearly the same for all the porous catalysts except for the one with 4.1 nm pores (as explained below). Configurational transport theory can predict the magnitude of the decrease in the observed *k*’s.

Assuming that our experimental system consists of cylindrical pores of a constant pore tortuosity containing uniform spherical AuNPs of diameter 4 nm, for a pore transport-limited reaction, the observed *k* should be related to the number of reaction sites (Γ′) times the effective pore diffusivity (*D*_e_),^54^ which is in turn proportional to other factors, as shown below
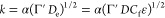
3where *D* is the bulk diffusivity of liquid *p*-nitrophenol, *C*_f_ is the configurational factor for small pores where the molecular size (0.66 × 0.43 nm elliptical cross section for *p*-nitrophenol)^55^ approaches that of the pore diameter, ε is the porosity, and Γ′ is the total number of the active sites which could be obtained by multiplying Γ by the average number of sites per AuNP. The porosity can be calculated as follows
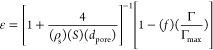
4where ρ_s_ is the skeletal density of the silica (used the value for quartz) and the other symbols have their previous meanings. The second bracketed term represents the fraction of pore volume occupied by the NPs themselves. The bulk diffusivity for *p*-nitrophenol in water^56^ and a *C*_f_ specifically developed for mesoporous silica in this size range^57^ were taken from the literature, and the normalized *k*’s were computed as shown in Figure 6g. Note that the number of AuNPs per pore (ψ) can also be calculated as
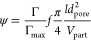
5

For 4.1 nm pores, ψ ∼ 2, and Γ in eq 3 was divided by 4 here because for 4.1 nm pores, a molecule of *p*-nitrophenol cannot pass the first AuNP; for one-way diffusion, both the second NP and the half of the first would be inaccessible. For all other pore sizes, passage is possible.

The agreement between the normalized (to *k* for the 4.1 nm pores) experimental *k*-values and the theory using eq 3 is acceptable considering the level of approximation involved. Complicating factors include the possibilities of slightly different NP shapes and sizes in the *m*SiO_2_, differing tortuosities, and effects of the AuNPs on the *C*_f_ function.

**Figure 7:**
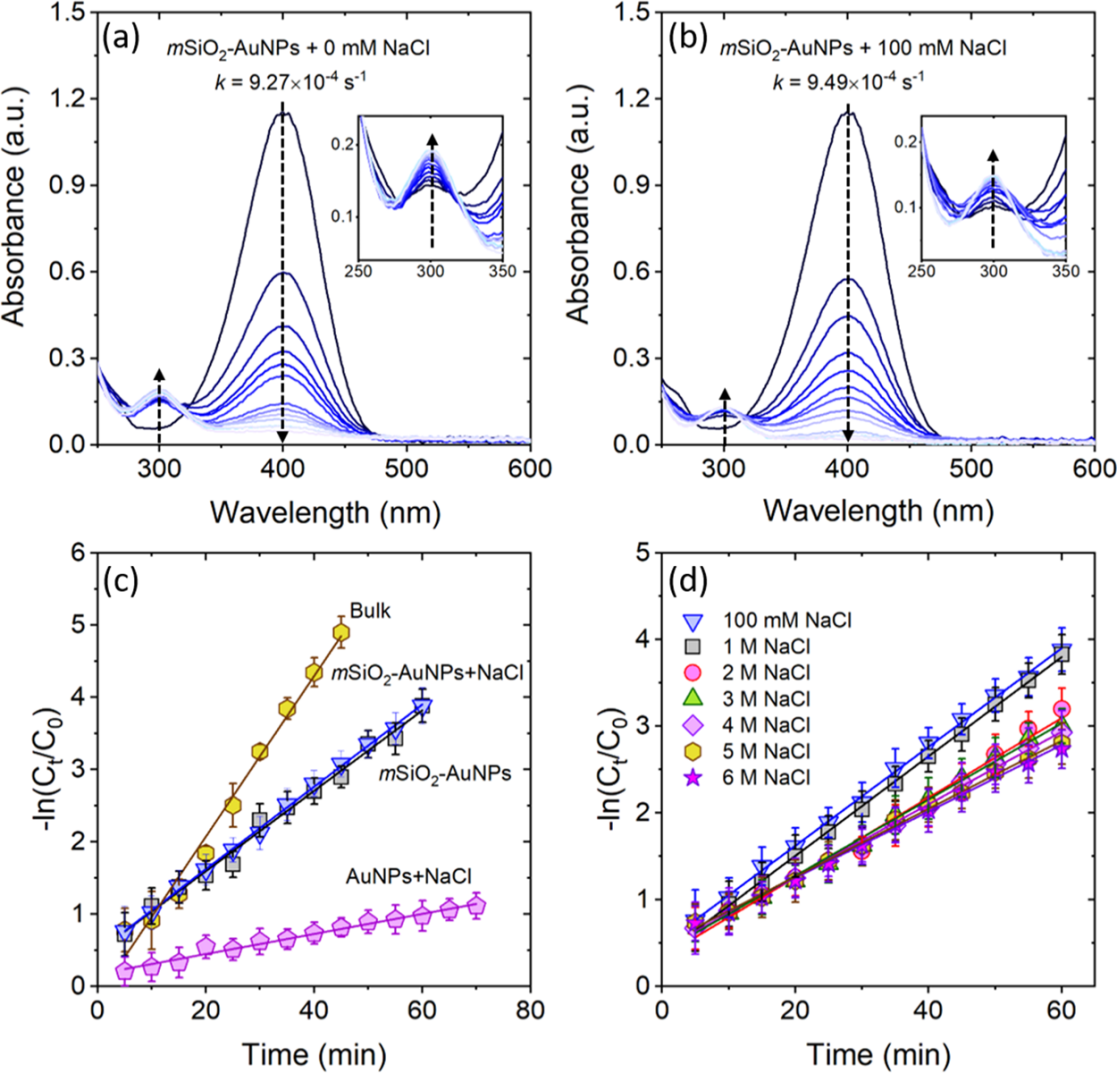
(a,b) Spectrophotometric profiles of the 4-nitrophenol reduction reaction using *m*SiO_2_–AuNP catalysts with and without 100 mM NaCl. The insets in (a,b) are the zoom-in plots of absorbance peak at 300 nm wavelength. Plots of −ln (*C*_*t*_/*C*_0_) as a function of time are for (c) AuNPs in bulk and *m*SiO_2_–AuNPs in the presence and absence of 100 mM NaCl and (d) *m*SiO_2_–AuNPs with increasing concentrations of NaCl.

Immobilizing AuNPs in *m*SiO_2_ maintains their spatially separated state in an extreme environment such as high salinity, allowing retention of catalytic activity. We monitor the change in the kinetics of the reaction upon the addition of NaCl to the aqueous solvent. The change −ln (*C*_*t*_/*C*_0_) with time for AuNPs dispersed in the solvent and immobilized in *m*SiO_2_ with *d*_pore_ = 8.4 nm in the presence and absence of 100 mM NaCl is shown in Figure 7a–c. Here, we represent the rate constants as *k*_AuNPs_ and *k*_*m*SiO_2_–AuNPs_, respectively, for dispersed and adsorbed (into *m*SiO_2_) AuNPs in DI water and *k*_AuNPs+NaCl_ and *k*_*m*SiO_2_–AuNPs+NaCl_, respectively, for dispersed and adsorbed AuNPs in water containing 100 mM NaCl. We find that the rate constant *k* follows the order: *k*_AuNPs+NaCl_ < *k*_*m*SiO_2_–AuNPs_ ≈ *k*_*m*SiO_2_–AuNPs+NaCl_ < *k*_AuNPs_. Because the number of AuNPs in all tested samples is identical, the catalytic activities are primarily dependent on the assembled state of AuNPs under different conditions. As discussed earlier, the screening of repulsions between the AuNPs upon the addition of electrolyte results in their aggregation. It is well-known that the number of active sites on AuNPs governs the reaction rate.^58^ The aggregation of AuNPs leads to a sharp decrease in the total surface area and the corresponding catalytically active sites exposed to the solvent, which leads to the observed decrease in the reaction rate. We find that *k*_*m*SiO_2_–AuNPs_ ≈ *k*_*m*SiO_2_–AuNPs+NaCl_, indicating that the adsorbed and spatially separated state of the AuNPs in nanopores is maintained upon the addition of 100 mM NaCl, which is also shown in the TEM and SANS studies (Figure 5). The spatially separated state of AuNPs in the pores is preserved up to 1 M NaCl, beyond which the rate constant shows a slight decrease (Figure 7d). At NaCl concentrations above 2 M, a small fraction of AuNPs likely desorb from the surface, leading to the observed slight decrease in their catalytic activity. Further studies are necessary to uncover the effect of such high concentrations of salt on the stability and reactivity of AuNPs in nanopores. Despite the minor decrease in the reaction rate, the experiments demonstrate that immobilizing the NPs in silica pores with a positive charge is a viable route to retain the catalytic activity of the AuNPs.

## 4 Conclusions

This study presented the effect of nanoconfinement on the adsorption and assembled state of AuNPs on amine-functionalized porous silica materials. We showed that the maximum amount of AuNPs that can be adsorbed on a silica matrix depends on the ratio of particle-to-pore dimaters. This increase results from the increase in the pore volume accessible to the AuNPs. The AuNP catalyst adsorbed in propylamine-modified mesoporous silica shows pore diameter-dependent reaction kinetics for the reduction of 4-nitrophenol to 4-aminophenol. Decreasing the pore diameter reduces the rate constant, due to transport limitations of reactants and products in and out of the pore space. We also demonstrated that the AuNPs retain their spatially distributed adsorbed state within the pores upon the addition of electrolyte, which leads to the preservation of their catalytic performance. The article helps in addressing a dichotomy on the pore size of inert supports to be used for catalysis. On the one hand, the decrease in pore diameter reduces the reaction rate; on the other hand, increasing the pore diameter results in a decrease in the specific surface area making the flat substrates impractical. For example, an ∼ 50 m^2^ silica wafer is required to drive the same reaction which can be performed with 1 g of *m*SiO_2_ with *d*_pore_ = 8.4 nm in a vial. This article provides the principle of finding an appropriate pore diameter of inert supports where the nanocatalyst can resist aggregation under extreme salinity environments while retaining a high catalytic activity and the corresponding reaction rates.

## Supporting Information Available

TEM images of AuNPs in bulk solution, nitrogen adsorption for different pore sizes of *m*SiO_2_, calibration curve of AuNPs for concentration determination, adsorption isotherms of AuNPs in different pore sizes of *m*SiO_2_ with the addition of salt, calibration curve of 4-nitrophenol in deionized water for the reduction reaction, derivation of the pore-filling fraction using the SVC model, centrifugation settling time calculation, details of DLVO theory, and model for analysis of SANS profiles (PDF)
