# Supplementary material for: Adsorption and Catalytic Activity of Gold Nanoparticles in Mesoporous Silica: Effect of Pore Size and Dispersion Salinity
Source: J Phys Chem C Nanomater Interfaces. 2022 Jan 26;126(5):2531–41. doi: 10.1021/acs.jpcc.1c09573 (PMC8842498; doi:10.1021/acs.jpcc.1c09573)
Supplement: Supplementary file 1 — jp1c09573_si_001.pdf [file jp1c09573_si_001.pdf]

Supporting Information

Adsorption and Catalytic Activity of Gold Nanoparticles in Mesoporous Silica: Effect of Pore Size and Dispersion Salinity

Yingzhen Ma[1], Gergely Nagy[2], Miriam Siebenbürger[3], Ravneet Kaur[4], Kerry M. Dooley[1], and Bhuvnesh Bharti[1,*]

[1]*Cain Department of Chemical Engineering, Louisiana State University, Baton Rouge, Louisiana 70803, USA*
[2]*Neutron Scattering Division, Oak Ridge National Laboratory, Oak Ridge, Tennessee, 37831, USA*
[3]*Center for Advanced Microstructures and Devices, Louisiana State University, Baton Rouge, Louisiana 70806, USA*
[4]*Life and Physical Science Department, Ivy Tech Community College of Indiana – Valparaiso, Indiana 46360, USA*

*Corresponding author's email: bbharti@lsu.edu

I. Transmission Electron Microscopy (TEM) images of AuNPs in deionized water

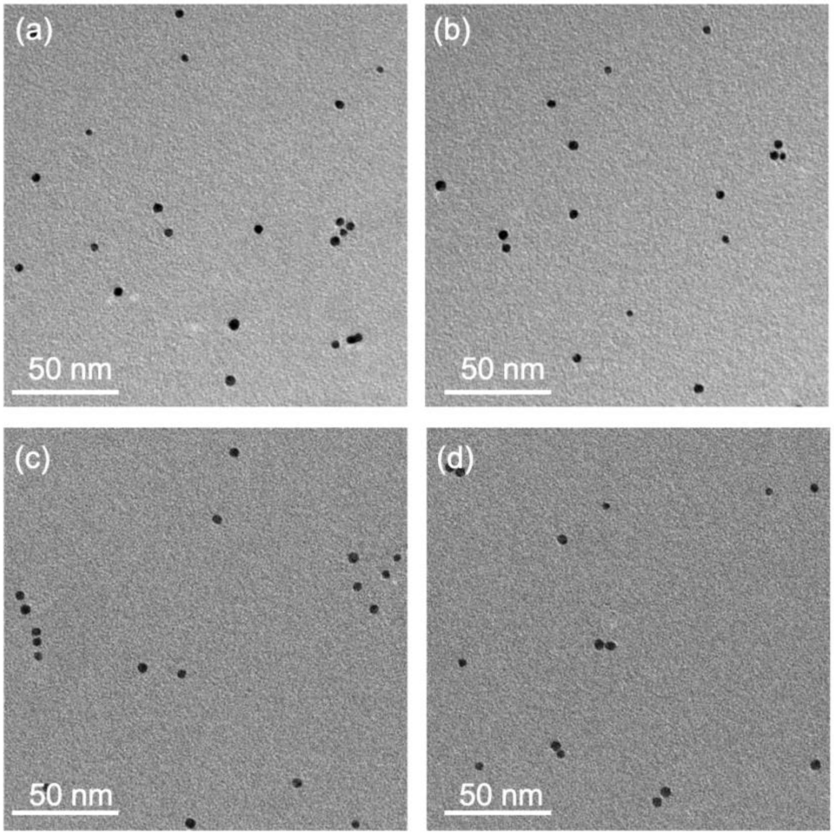

Figure S1. (a-d) TEM images of AuNPs in DI water, which shows the average diameter of AuNPs in solution is about 4 nm.

II. Nitrogen adsorption for different pore size of *m*SiO_2

The average pore diameter of functionalized MCM-41 and SBA-15 mesoporous silica was characterized using nitrogen gas adsorption (Micromeritics, ASAP 2020).

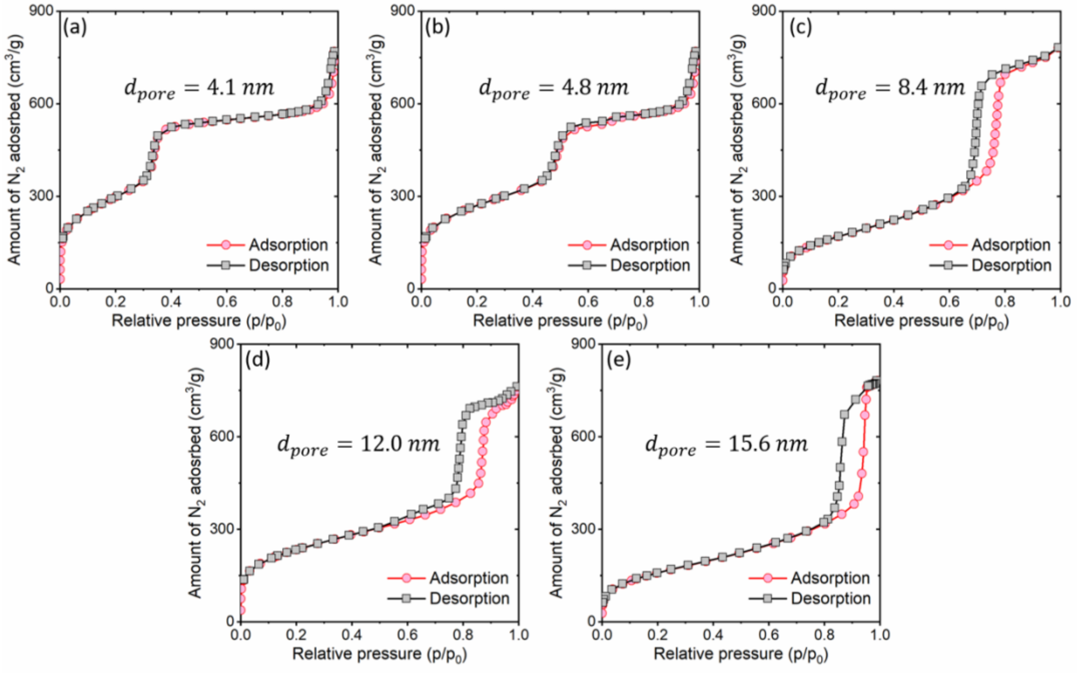

Figure S2. Nitrogen gas adsorption isotherm for different pore diameter of *m*SiO_2 showing a characteristic adsorption−desorption hysteresis.

Here, the average pore diameter is calculated from the the relative pressure (p/p_0) at the capillary condensation step of the isotherm using modified Kruk−Jaroniec−Sayari (KJS) model, which is given as (expressed in nm)[1]

$$d_{pore} = \frac{-1.15}{\log\left(\frac{0.875\mathrm{p}}{p_0}\right)} + 0.2\left[\frac{60.65}{0.03 - \log\left(\frac{p}{p_0}\right)}\right]^{0.397} + 0.27$$

III. Calibration curve of AuNPs in bulk solution for adsorption isotherms

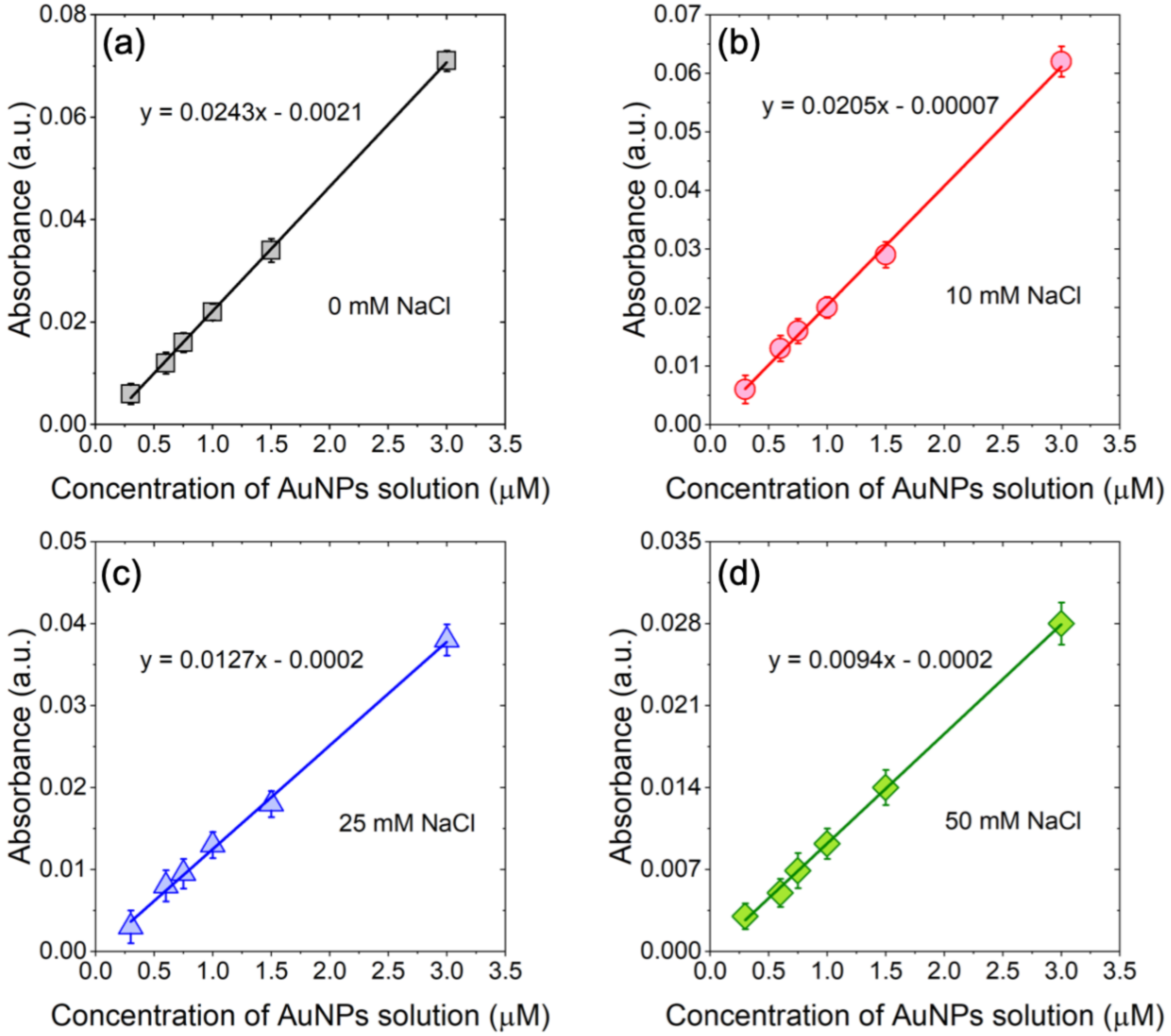

Figure S3. The spectrophotometric absorbance calibration curve of AuNPs in bulk solution. The symbols are the experimentally measured absorbance values of a given concentration of AuNPs, and the lines are the linear fits to the experimental data. The equation of line representing the value of absorbance increases as increasing concentration of AuNPs, where x and y are the concentration of AuNPs in bulk and corresponding absorbance value, respectively.

IV. Pre-adsorption isotherms of AuNPs in different pore size *m*SiO_2 with addition of salt

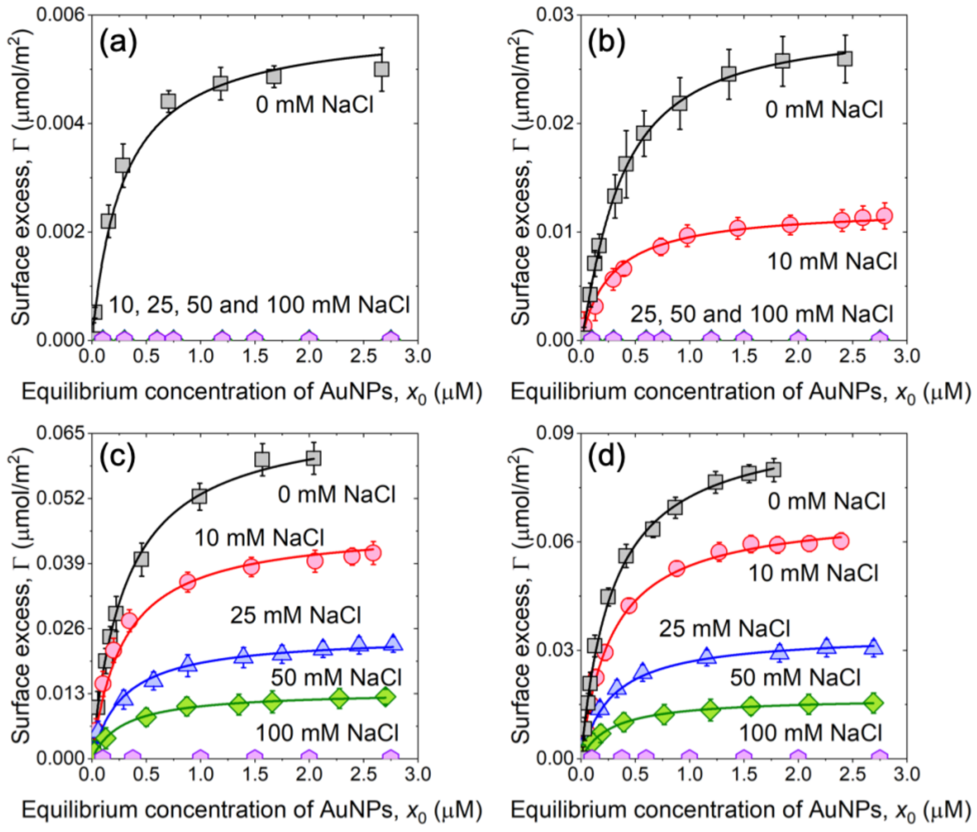

Figure S4. Adsorption isotherms of AuNPs on *m*SiO_2 with (a) 4.1 nm, (b) 4.8 nm, (c) 12.0 nm, and (d) 15.6 nm pore diameters in the presence of 0, 10, 25, 50 and 100 mM NaCl. Here the NaCl was added to the AuNPs dispersion prior to their adsorption into *m*SiO_2. The discrete points are experimental values, and solid lines are fit using Langmuir model.

V. **Post-adsorption isotherms of AuNPs in different pore size *m*SiO_2 with addition of salt**

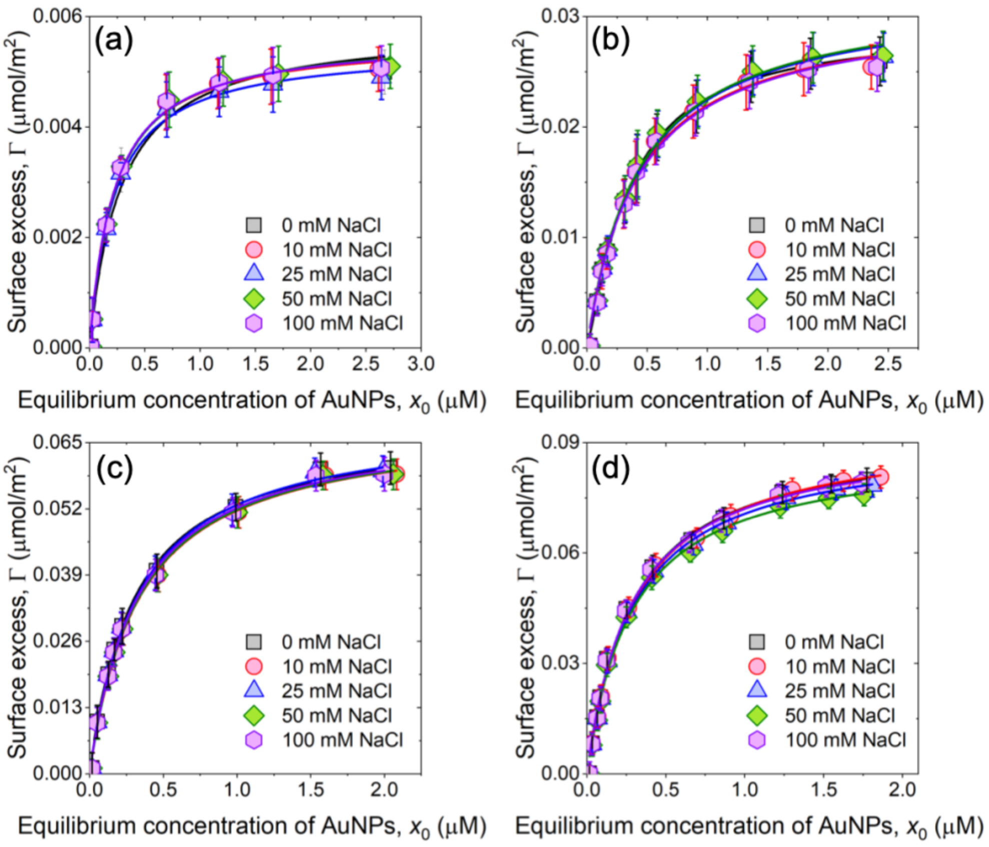

Figure S5. Adsorption isotherms of AuNPs in *m*SiO_2 with (a) 4.1 nm, (b) 4.8 nm, (c) 12.0 nm, and (d) 15.6 nm pore diameters with increasing concentration of NaCl. The electrolyte was added to the dispersion after AuNPs were adsorbed within the pores of *m*SiO_2. The points are the measured experimental values, and solid lines are represented using Langmuir model.

VI. Calibration curve of 4-nitrophenol in deionized water for the reduction reaction

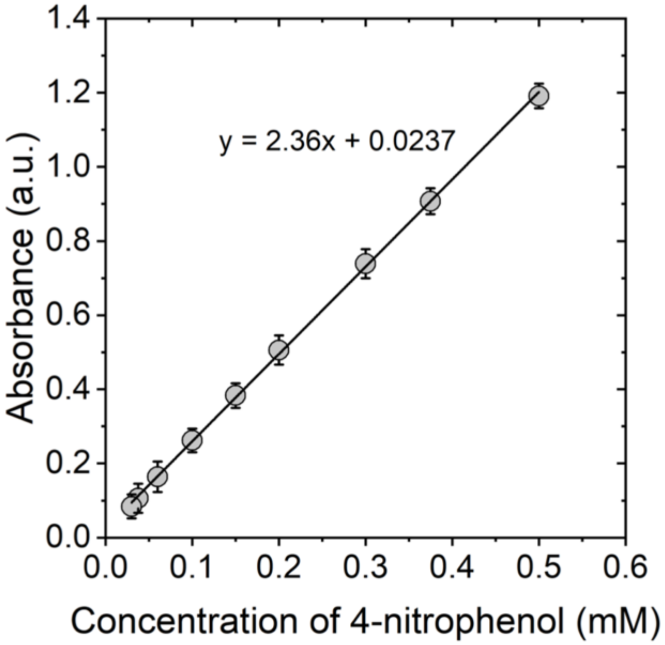

Figure S6. The absorbance calibration curve of 4-nitrophenol in deionized water. The black circles are the absorbance values of known concentrations of 4-nitrophenol, and the black line is the linear representative to the experimental values. The equation of the trendline shows an increase absorbance value as increasing concentration of 4-nitrophenol, where y and x represent the absorbance value and the concentration of 4-nitrophenol in deionized water, respectively.

VII. Settling time calculation

We estimated the settling time (t) of AuNPs + mSiO$_2$ and free AuNPs using Stokes Law at $18{,}000g$ as:

$$\mathrm{t} = \frac{18\mu L'}{(\rho_p - \rho_w)g' d^2}$$

where μ is dynamic viscosity of water at 20 °C, L' is the length of centrifuge vial, ρ_p and ρ_w are the densities of AuNPs or mSiO$_2$ and water, respectively, g' is the centrifugal field strength $(18{,}000g)$, g is the gravitational acceleration and d is the diameter of nanoparticles. Here we assumed mSiO$_2$ as spheres of diameter 100 nm and AuNPs as spheres of diameter 4 nm. The settling time for free AuNPs is also calculated in the similar manner. The settling time of mSiO$_2$ under $18{,}000g$ is 6 minutes and for AuNPs is 226 minutes. Thus, the samples were centrifuged for 30 minutes to separate mSiO$_2$ with adsorbed AuNPs from free AuNPs.

VIII. Derivation of pore filling fraction using SVC model

The maximum amount of AuNPs can be arranged in pores with cross-section area $A_{pore} = (\pi/4)\, d_{pore}^2$ is given[2,3]

$$n_{max} = \frac{N}{N_A A_{pore}\sigma} V_{pore} \tag{1}$$

where N is the number of molecules that can be accommodated side-by-side in a cross-section of the pore, N_A is the Avogadro constant, σ is the diameter of AuNPs, and V_{pore} is the specific pore volume of the mSiO2. Here, a 2D model of AuNPs packing is applied without consideration of packing effects in the direction of the pore axis in equation (1). The volume fraction of AuNPs is represented by

$$f = \frac{N V_{AuNPs}}{(\pi/4) d_{pore}^2\, \sigma} \tag{2}$$

where V_{AuNPs} is the volume of an AuNP, and $V_{AuNPs} = \pi\sigma^3/6$. Plugging this into equation (2) and combing with equation (1) gives

$$n_{max} = \frac{6}{N_A \pi \sigma^3} f V_{pore} \tag{3}$$

By introducing the pore size distribution function $M(d_{pore}) = (dV_{pore}/d(d_{pore}))_{d_{pore}}$, given

$$n_{max} = \frac{6}{N_A \pi \sigma^3} \int f(d_{pore})\, M(d_{pore}) d(d_{pore}) \tag{4}$$

with

$$f = \frac{2N}{3(d_{pore}/\sigma)^2} \tag{5}$$

where the integral extends over the relevant pore size. Depending on ratio of d_{pore}/σ, the N value is obtained by

$$N(d_{pore}/\sigma) = \begin{cases} 0 & d_{pore}/\sigma < 1 \\ 1 & 1 < d_{pore}/\sigma < 2 \\ N_1 & 2 < d_{pore}/\sigma < 3 \\ N_1 + 1 & 3 < d_{pore}/\sigma < 4 \end{cases} \tag{6}$$

with

$$N_1 = \pi \left(arcsin \frac{\sigma}{d_{pore} - \sigma} \right)^{-1} \tag{7}$$

IX. DLVO theory

According to DLVO theory, the net interaction energy between colloidal particles (U^{DLVO}) dispersed in an aqueous medium is the sum of electrostatic (U^{Elec}) and van der Waals (U^{vdw}) interactions given as[4]

$$U^{DLVO} = U^{Elec} + U^{vdw} \tag{8}$$

For a pair of spherical AuNPs of radius R, the electrostatic and van der Waals interaction energy is estimated using following equations[4]

$$U^{Elec}_{AuNP-AuNP} = 32\pi\varepsilon_0\varepsilon_r\left(\frac{k_BT}{e}\right)^2 R\tanh^2\left(\frac{e\phi_0}{4k_BT}\right)\exp\left(-\kappa h\right) \tag{9}$$

and

$$U^{vdw}_{AuNP-AuNP} = \frac{-AR}{12h} \tag{10}$$

where $U^{Elec}_{AuNP-AuNP}$ and $U^{vdw}_{AuNP-AuNP}$ are respectively the electrostatic and van der Waals interactions, ε_0 is the permittivity of vacuum, ε_r is the dielectric constant of the medium, k_B is Boltzmann constant, T is the temperature, e is the electric charge, ϕ_0 is the surface potential of the interacting particles, κ is the inverse of Debye length, h is the distance between the colloidal particles, and A is the Hamaker constant of the colloidal particles. In our case for simplicity reasons, we assume the surface potential ϕ_0 to be equal to the zeta potential of sodium citrate-capped AuNPs in dispersion. For calculating the interaction between the AuNPs and tubular silica pore, we approximate the pore-wall as a flat surface and the DLVO interaction energy between a particle and a plane is estimated as[4,5]

$$U^{Elec}_{mSiO_2-AuNP} = \pi\varepsilon_0\varepsilon_r R\left[2\phi_0\phi_1 ln\left(\frac{1+\exp\left(-\kappa h\right)}{1-\exp\left(-\kappa h\right)}\right) + (\phi_0^2+\phi_1^2)\ln\left(1-\exp\left(-2\kappa h\right)\right)\right] \tag{11}$$

and

$$U^{vdw}_{mSiO_2-AuNP} = \frac{-AR}{6h} \tag{12}$$

where $U^{Elec}_{mSiO_2-AuNP}$ and $U^{vdw}_{mSiO_2-AuNP}$ are respectively the electrostatic and van der Waals interactions between an AuNP and mSiO$_2$ flat surface, and ϕ_1 is the surface potential of mSiO$_2$. Here we assume that the surface potential of mSiO$_2$ is equal to the zeta potential of propylamine-modified nanoparticles, reported in our previous study.[6]

X. Model for analysis of SANS profiles

The total scattering intensity (I_{total}) of AuNPs in the mSiO$_2$ pores under contrast matched condition is given as[7]

$$I_{total}(q) = I_{Bragg}(q) + I_{Diff}(q) \quad (13)$$

where $I_{Bragg}(q)$ is the Bragg scattering contribution from the pore lattice with adsorbate (AuNPs), and $I_{Diff}(q)$ is the diffuse scattering attributed to the inhomogeneous distribution of the scattering objects within the cylindrical pores and outside the pores if unadsorbed objects exist. The diffuse scattering contribution is modeled using the Teubner-Strey function

$$I_{Diff}(q) = \frac{I_0}{\left(1-\frac{I_0}{I_m}\right)\left(\frac{q^2}{q_m^2}-1\right)^2+\frac{I_0}{I_m}} \quad (14)$$

where I_m and q_m represent the coordinates of the maximum in the correlation peak, and I_0 is the forward scattering intensity. The quasiperiodic distance (M) between AuNPs is calculated from I_m, q_m and I_0 in eq. (15) as

$$M = 2\pi\frac{\sqrt{2}}{q_m}\left[\left(\frac{I_m}{I_0}\right)^{1/2} + 1\right]^{-1/2} \quad (15)$$

The Bragg scattering contribution is given as

$$I_{Bragg}(q) = \frac{nN_{pore}}{V}V_{part}^2(\Delta\rho^2)P(q)S(q) \quad (16)$$

with

$$P(q) = \left[\frac{3(sin(qR)-(qR)cos(qR))}{(qR)^3}\right]^2 \quad (17)$$

where n is the number of AuNPs in each cylindrical pore, N_{pore} is the total number of cylindrical pores in the scattering volume $V = \frac{3\sqrt{3}}{2}H^2 l$ with l being the length of the pore, and $H = md_{pore} + (m-1)(l_p - d_{pore})$, where m is the order number of the 2D *hcp* porous matrix, d_{pore} is the pore diameter, and l_p is the lattice parameter. V_{Part} is the volume of the single scattering object/particle, $\Delta\rho$ is the scattering length density contrast between the scattering object and the solvent, $S(q)$ is the structure factor, $P(q)$ is the form factor of the scattering object, and R is the mean radius of AuNPs.

References

(1) Jaroniec, M.; Solovyov, L. A. Improvement of the Kruk−Jaroniec−Sayari Method for Pore Size Analysis of Ordered Silicas with Cylindrical Mesopores. *Langmuir* **2006**, *22* (16), 6757–6760.

(2) Meissner, J.; Prause, A.; Di Tommaso, C.; Bharti, B.; Findenegg, G. H. Protein Immobilization in Surface-Functionalized SBA-15: Predicting the Uptake Capacity from the Pore Structure. *J. Phys. Chem. C* **2015**, *119* (5), 2438–2446.

(3) Sang, L. C.; Vinu, A.; Coppens, M. O. General Description of the Adsorption of Proteins at Their Iso-Electric Point in Nanoporous Materials. *Langmuir* **2011**, *27* (22), 13828–13837.

(4) Israelachvili, J. N. *Intermolecular and Surface Forces*, 3rd ed.; Elsevier B.V., 2011.

(5) Hogg, R.; Healy, T. W.; Fuerstenau, D. W. Mutual Coagulation of Colloidal Dispersions. *Trans. Faraday Soc.* **1966**, *62* (615), 1638–1651.

(6) Ma, Y.; Wu, Y.; Lee, J. G.; He, L.; Rother, G.; Fameau, A.-L.; Shelton, W. A.; Bharti, B. Adsorption of Fatty Acid Molecules on Amine-Functionalized Silica Nanoparticles: Surface Organization and Foam Stability. *Langmuir* **2020**, *36* (14), 3703–3712.

(7) Ma, Y.; Heller, W. T.; He, L.; Shelton, W. A.; Rother, G.; Bharti, B. Characterisation of Nano-Assemblies inside Mesopores Using Neutron Scattering*. *Mol. Phys.* **2021**, *e1905190*.
